# The Spectrum of Heat-Related Diseases - A Meta-Review

**DOI:** 10.3389/ijph.2025.1608592

**Published:** 2025-09-16

**Authors:** J. Thiel, A. Seim, B. Stephan, M. Sedlmayr, E. Prochaska, E. Henke

**Affiliations:** Institute for Medical Informatics and Biometry, Faculty of Medicine and University Hospital Carl Gustav Carus, TUD Dresden University of Technology, Dresden, Germany

**Keywords:** review, climate change, global warming, heat-related diseases, climatic factors

## Abstract

**Objectives:**

Global warming affects health and puts a strain on the healthcare system. Prediction models can forecast healthcare demand and optimize resource allocation. The aim of this study was to identify heat-related diseases and their influencing factors necessary for the development of such a prediction model.

**Methods:**

A literature search was conducted in the PubMed, Embase and Medline databases. The focus was on reviews of heat-related diseases published within the past 30 years in the German and English languages. A qualitative synthesis of the results was conducted.

**Results:**

The literature search produced a total of 737 results. A total of 15 reviews were included in the synthesis. As a result of the synthesis, a spectrum of heat-related diseases such as electrolyte imbalances, cardiovascular disease, kidney disease, respiratory disease, mental health issues, infectious diseases and other diseases were identified. Furthermore, specific climatic and other influencing factors were determined.

**Conclusion:**

The meta-review highlighted a wide range of diseases that can occur in connection with heat, along with their influencing factors. The findings can serve as the basis for developing preventive measures such as a prediction model in order to better forecast the resource load due to acute heat.

## Introduction

A global rise in temperature is predicted due to climate change, as a result of which the intensity and duration of heatwaves are also likely to increase [1]. The consequences for human health are manifold and range from various illnesses to death [2]. A key challenge of climate change is coping with rising temperatures and more frequent heatwaves, which are associated with an increased mortality rate [3]. Heat-related deaths result from a combination of heat exposure and pre-existing conditions, which makes it difficult to accurately assess health impacts and develop effective prevention strategies [4]. As a result of climate change, the World Health Organization (WHO) predicts up to 250,000 additional deaths between 2030 and 2050 [1]. Heatwaves can lead to more hospital admissions [5, 6] and therefore place an additional burden on the healthcare system [7] and on the healthcare staff [8]. This can increase the risk of capacity problems [9].

In the literature, heat diseases are defined as conditions that are the direct result of climatically induced ambient heat in combination with a disturbance of thermoregulation [1, 10]. Heat-related health problems are manifold and manifest themselves not only as typical heat diseases such as fluid balance disorders or heat stroke [11], but also as an increased risk of cardiovascular diseases such as heart attacks or heart failure [11], respiratory diseases such as asthma or chronic obstructive pulmonary disease (COPD) [12] and others. The WHO has emphasized that, in addition to global climate protection, targeted measures to protect the population from heat-related health risks are also essential [7]. In 2017, the German Federal Ministry for the Environment, Nature Conservation, Building and Nuclear Safety issued recommendations for the development of heat action plans to protect public health. These plans should be implemented at both the state and municipal levels [13, 14]. Approaches to this can be seen, for example, in the United Kingdom, where plans for various heat protection measures are implemented in the summer depending on the temperature [15]. The literature also shows that the prediction of heat-related patient admissions is a possible approach [16].

In this context, digital tools could play a valuable role [17]. These could warn both the public and healthcare professionals about the specific effects of heat events [17]. However, such digital tools are currently hardly used in Germany, which means that their potential remains untapped [17]. In order to optimize the digital transformation of the German healthcare system, the Federal Ministry of Education and Research is funding initiatives such as the Medical Informatics Initiative and six Digital Health Progress Hubs. One of these initiatives, the Medical Informatics Hub in Saxony (MiHUBx), aims to develop a predictive tool for hospital utilization during acute heat events in Saxony [18].

Various exposure, care and modeling data are required for the development of such tools [19]. These include, for example, weather data such as temperature and cloud cover, morbidity data in the observed population, and the socio-demographic characteristics of the same population [19]. First an overview of diseases associated with heat is required in order to be able to investigate and quantify heat-related morbidity in the population. Existing reviews often focus on individual risk groups such as outdoor workers [20] or are very disease-specific, as they are focused on specific diseases such as kidney disease [21], mental health issues [22] or cardiorespiratory diseases [23]. However, we are not aware of any work that provides a comprehensive picture of all possible heat-related diseases. Consequently, there is currently a lack of a comprehensive basis that is specifically required for the development of a prediction model.

The aim of this study is to address this research gap and lay the groundwork for the development of a prediction model for the utilization of the healthcare system during acute heat events. Developing an effective prediction model first requires a deeper understanding of the relationship between acute heat and specific diseases in the study area. To our knowledge, there is insufficient empirical evidence on the relationships between specific diseases and the utilization of healthcare services, particularly in German hospitals and especially in Saxony. Only national and state-specific analyses of individual, selected diseases are available, to our knowledge [24, 25]. Region-specific analyses, which enable small-scale predictions and measures, are lacking.

This work is intended to provide a broad overview of the relevant data, particularly heat-related diseases, in order to be able to examine the relationship between heat exposure and disease development in more detail in the future. This should enable the investigation of correlations between heat and resource utilization in the healthcare system in general, but especially in Saxony, and thus also create a basis for the development of region-specific prediction models.

## Methods

### Literature Search

To obtain a comprehensive overview of existing heat-related diseases, the specific search object was first defined using the *Population*, *Exposure*, *Comparison*, *Outcome* and *Study Design* (PECOS) framework. Furthermore, concrete inclusion and exclusion criteria were defined (Table 1). Subsequently, scientific literature was used to identify relevant search terms. The individual search terms were combined using Boolean operators according to the syntax of the databases used: PubMed (accessed via PubMed), Embase and Medline (both accessed via OVID). The search strings were subsequently tested in the respective databases and iteratively optimized until the desired results were obtained. The final search strings for PubMed and OVID are provided in the Supplementary Appendix SA1. To ensure quality, the manuscript was checked using the PRISMA (Preferred Reporting Items for Systematic reviews and Meta-Analyses) checklist after completion of the meta-review [26].

**TABLE 1:** Inclusion and exclusion criteria (Dresden, Germany, 2024).

Category	Inclusion criteria	Exclusion criteria
Patient/Population	Human population (regardless of age, gender, or pre-existing conditions)	Animals
Exposure	Weather-related heat exposure	No heat exposure or no weather-related exposure (e.g., saunas, Occupational exposure such as from welding)
Comparison	Varying degrees of heat exposure, influence of additional factors	-
Outcome	Diseases identified in connection with heat exposure	Other outcomes besides heat-related diseases
Study Design	Published reviews/meta-analyses in German/English	Pre-prints, non-reviews/non-meta-analyses (comments, primary studies, etc.), and different languages

### Synthesis of Results

The data analysis process was conducted by five researchers from the fields of public health, sociology and medical informatics at the Institute for Medical Informatics and Biometry at the Technical University of Dresden. The search strings were then applied to the databases on 15 May 2024. The resulting studies were exported and afterwards imported into the web-based collaboration tool Rayyan [27]. Rayyan automatically screened the imported studies for duplicates. One of the reviewers manually verified that the flagged studies were indeed duplicates and removed them accordingly. For the remaining studies, a title-abstract screening (TAS) was subsequently performed. To ensure TAS quality, 20 studies were independently screened by all reviewers, who discussed their decisions. The remaining studies were then divided among the reviewers and a full TAS was performed. The studies were sorted alphabetically by author, and each reviewer was assigned a quarter of them. One reviewer assessed all included studies. This was conducted in blind mode, whereby each study was always screened independently by two reviewers. In case of conflicts, the respective study was discussed and if necessary, a third reviewer was involved to resolve it.

Next, the studies that met the inclusion criteria in the TAS underwent a full-text screening (FTS), following the same process as the TAS. Finally, an analysis of the full texts and extraction of relevant results was using a pre-designed extraction table. During data extraction, two reviewers analyzed the studies independently. The individual tables of the reviewers were then consolidated into a single results table. Information on the individual diseases, the study population, climatic factors influencing disease development and other influencing factors was extracted. To ensure the accuracy and completeness of the data, this consolidated table was reviewed and verified by all reviewers.

## Results

### Identified Studies

**FIGURE 1:**
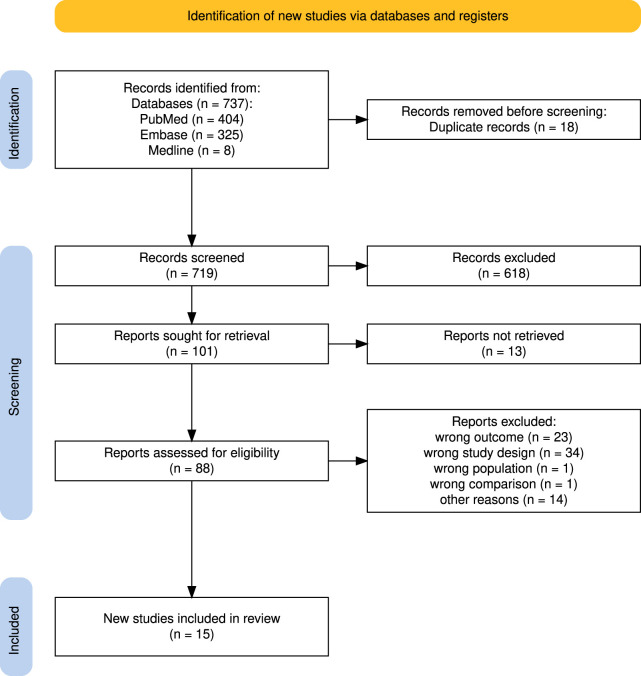
Flowchart with an overview of the identified, included and excluded studies (Dresden, Germany, 2024) [36].

A total of 737 search results (PubMed: 404; OVID: 333) were retrieved. After removing duplicates, 719 studies were included in the TAS. The majority of exclusions (618) occurred during the TAS. During the FTS, a further 86 studies were excluded due to missing full texts (13) or not meeting the inclusion criteria (73). Finally, 15 studies, published between 1998 and 2024, were included in the final analysis [10–12, 20–23, 28–35]. A detailed overview of the number of studies included and excluded during the analysis process can be found in the PRISMA flowchart (Figure 1).

### Characteristics of the Included Reviews

All articles reported on heat-associated diseases, climate-related factors, and general risk factors. Nine of the 15 included studies did not focus on a specific population group [11, 12, 21, 23, 29–31, 34, 35]. The remaining six works examined specific risk groups such as athletes, outdoor workers, diabetics, intensive care patients and individuals with mental conditions [10, 20, 22, 28, 32, 33]. There were no restrictions on the study population based on sociodemographic characteristics. All other included articles were published within the last 10 years from the start of the search, except for the one by Noakes (1998) [33].

### Identified Heat-Related Diseases

The articles provided a general overview of heat-related diseases. These were reported as free text or as diagnostic codes from the “International Statistical Classification of Diseases and Related Health Problems” (ICD), versions 9 or 10, in addition to a combination of free-text and ICD codes. Overall, the results of the review demonstrate that heat exposure can affect a wide range of acute and chronic diseases, with health consequences that can be both physical and psychological. The following section describes the identified diseases:

Heat-Specific Diseases: Heat-specific diseases occur exclusively due to heat exposure and include heat cramps, heat collapse (also known as heat exhaustion or heat syncope) and heat stroke [10–12, 21–23, 28–30, 34, 35].

Water and Electrolyte Imbalances: Among the most commonly reported conditions related to heat were water and electrolyte imbalances, such as dehydration or hypernatremia [28, 33, 35].

Cardiovascular Diseases: Heat exposure increases the risk of cardiovascular diseases, including heart attack (myocardial infarction), heart failure and heart stroke. Acute coronary syndrome and out-of-hospital cardiac arrest have also been associated with periods of extreme heat [11, 21, 23, 32].

Kidney Diseases: Both acute and chronic kidney damage and diseases have been reported in association with heat exposure [11, 21, 30].

Respiratory Diseases: Respiratory conditions, such as chronic obstructive pulmonary disease (COPD), asthma and acute respiratory failure, often worsen during periods of high temperatures [23, 30, 32].

Mental Health Diseases: Heat exposure is also linked to mental health conditions such as schizophrenia and other disorders, including bipolar disorder, dysthymia and depressive disorders [22].

Infectious Diseases: Acute heat events can also influence the prevalence of infectious diseases such as malaria, along with respiratory infections such as pneumonia [30].

Other Diseases: Additional conditions identified in this meta-review include fatigue and general diarrheal diseases. Notably, there is an increase in heat-stress-related accidents, such as exhaustion and loss of consciousness [10, 20].

### Climatic Influencing Factors

As part of the meta-review, various climatic factors were identified that are associated with the occurrence of heat-related diseases - these are shown in detail in Supplementary Appendix SA2. Temperature was consistently mentioned as a key factor, playing a central role in all the studies considered. In addition, (relative) humidity and in some cases air pressure, wind movement, and direct sunlight were described as climatic influences [10–12, 20, 28–30, 32–35]. Several studies referred to combinations of these factors, such as temperature and humidity or temperature, humidity and wind movement. Furthermore, air quality or air pollution in combination with temperature was also considered an additional influencing factor in some studies. Other reviews reported the use of a specific heat index in studies, designed to better account for the combination of these individual factors and their possible interaction effects between environmental factors [10, 20, 32, 33].

### Other Influencing Factors

In addition to climatic conditions, a variety of other factors influencing the risk of heat-related diseases were identified in the analyzed studies (see Supplementary Appendix SA2). Frequently mentioned risk factors include age (particularly individuals over 65 years and children) and gender (predominantly male). Furthermore, individual characteristics and behaviors were described as risk factors, such as existing or impending dehydration, sweating, intense physical activity (e.g., physical labor), lack of acclimatization and wearing weather-inappropriate clothing. It was also found that pre-existing conditions, such as cardiovascular diseases, diabetes mellitus, skin abnormalities, and conditions such as chronic kidney disease or mental disorders, increase susceptibility to heat-related diseases. Additionally, factors such as low socioeconomic status, lack of access to fluids, insufficient rest breaks and inadequate sun protection were extracted as risk factors from the included reviews. Other risk factors include drug and alcohol use, medication intake, previous heat-related injuries, and increased physical strain due to manual labor or intense athletic activities - particularly in the sun and without sufficient hydration and breaks [10–12, 20–23, 28–35].

## Discussion

The meta-review provided a comprehensive overview of heat diseases supported by a high level of evidence. The study highlighted the wide range of heat-related diseases, from those specific to heat exposure to infections and mental health conditions. While many of these conditions are not exclusively caused by heat, heat exposure often acts as a contributing factor within a multicausal framework. This should be taken into account when examining these diseases and calls for further research to investigate and quantify their exact influence on pathogenesis.

A similar meta-review- that provides an overiew of heat-related diseases could not be found in the preliminary work for this meta-review. It is therefore difficult to classify it in the research context. The meta-review is particularly characterized by its general broad overview, which is also reflected in the diversity of the results. The included reviews often only provided an overview of one or more risk groups, such as patients in intensive care [28], mental diseases [22] or cardiorespiratory effects [23]. The partly emerging heterogeneity of the included studies also revealed problems related to the lack of standardization with the type of reporting of diseases (different ICD versions and/or free text).

The majority of reviews identified male gender as a risk factor for heat-related diseases, as reported by study teams led by Cheng, Santelli, Gauer, Liu, Wu and Moon [10, 21, 23, 32, 34, 35]. However, Xu et al. found that the female gender posed a risk [11], while Arsad et al. reported an elevated risk for both genders [12]. Although no definitive conclusion regarding gender can be drawn, the male gender was more frequently reported as a risk factor. Age was consistently recognized as a risk factor, with both children and the elderly being particularly vulnerable. This aligns with the recommendations from the WHO’s heat action plans, which emphasize protecting these populations [7]. These findings are also consistent with research on other heat-associated conditions, such as asthma [37, 38] and diabetes [39], where both extremes of age are recognized as risk factors. Socioeconomic status similarly emerged as a key risk factor, reflecting WHO recommendations and broader scientific discussions [7]. For example, lower-income populations may live in hotter environments or may have difficulty accessing adequate cooling measures, such as air conditioning [40, 41].

This study also suggested possible interaction effects between different diseases. For example, established associations between heat exposure, dehydration and diseases such as cardiovascular conditions, as discussed by Watso et al. [42], were reinforced by this review. However, health effects directly attributed to solar radiation, such as sunburn, were excluded. This highlights the specificity of the search strategy, which effectively isolated heat-related outcomes while minimizing irrelevant findings. Furthermore, this emphasizes the importance of systematically capturing the health impacts of heat and potential adaptation strategies. A conclusion that was also drawn in previous reviews on heat-related health outcomes and adaptation measures [43]. Nevertheless, in future models that account for pre-existing conditions, such damages due to solar radiation should be considered, as they contribute to the seasonal burden on healthcare systems.

In addition to analyzing the associations between heat and the identified health conditions, future research should place greater emphasis on potential adaptation strategies and their impact on morbidity. Initial evidence suggests that the implementation of such strategies may contribute to a reduction in heat-related health outcomes. However, it remains unclear which specific measures are the most effective, due in part to the multifactorial nature of the underlying mechanisms and the complexity of contributing factors. Accordingly, future studies should aim to identify and analyze the key influencing factors of specific conditions and evaluate adaptation strategies within their respective regional contexts [43, 44].

### Strengths and Limitations

The meta-review summarized the results of studies on heat-related diseases conducted over the last 30 years, providing a comprehensive overview with a high level of evidence. However, it was also shown that the exact quantification of existing relationships is lacking. This also led to a limitation of the study, which is that no exact statements can be made about the influence of heat on pathogenesis. In addition, it was not possible to estimate the exact influence of heat or other factors on disease manifestation. It should be noted that the quality of the individual reviews was not assessed separately. This is often recommended in the literature [45] in order to assess the significance of the individual studies and to incorporate their results differently based on the quality of the synthesis. As this study only included reviews that had already been published and subjected to quality checks, and a purely qualitative overview of existing diseases was compiled, the risk of bias was rather low. Nevertheless, it cannot be ruled out that certain groups of people may be over- or underrepresented. In addition, a high degree of heterogeneity was shown with regard to the use of different ICD versions and variants or even the use of free text, which makes it difficult to compare the studies with each other. Semantic standardization is a prerequisite for conducting detailed statistical analyze. Additionally, there was a potential for duplication of primary studies across different reviews, although this was unlikely to introduce significant bias, as no quantitative effect sizes were calculated in this work. It should also be noted that no publicly accessible protocol was created for this study, nor was it registered in an appropriate database, which could potentially impact the transparency of the research process. However, this did not necessarily diminish the quality of this work, as all steps were documented in a comprehensible and transparent manner.

### Conclusion

The initial objective of the meta-review - to create a comprehensive overview of heat-related diseases - was achieved, providing a foundation for the development of prediction models for acute heat events. A wide range of diseases was identified, including not only heat-related conditions such as heat stroke, but also other diseases such as electrolyte imbalances, cardiovascular and respiratory diseases, kidney diseases and mental health conditions, along with an increased incidence of some infectious diseases. It is important to emphasize that these diseases are influenced by more than just heat. Through the review, it was possible to identify climatic factors such as temperature and humidity and other influencing factors such as age, gender and socioeconomic status.

In conclusion, this work provides a broad overview of diseases that can occur in the context of heat exposure. This overview can serve as a basis for further research and the development of specific measures. Therefore, our work provides a promising step to support the development of forecast models and the targeted design of heat action plans, in addition to general preventive measures. However, further research is needed, particularly regarding the quantification of the exact influence of heat on the development of the identified diseases. In this context, the climatic and other influencing factors highlighted in the meta-review should also be explicitly considered.

## References

[1] World Health Organization. Climate Change (2023). Available online at: https://www.who.int/news-room/fact-sheets/detail/climate-change-and-health (Accessed 2024 February 8).

[2] Saravanan S Akay YM Chen T Akay M. Impacts of Climate Change on Global Health: A Review of Preparedness, Infectious Disease, and Excessive Heat. Health Technol (2025) 15(1):7–14. 10.1007/s12553-024-00927-7

[3] Winklmayr C Muthers S Niemann H Mücke HG An Der Heiden M. Heat-Related Mortality in Germany from 1992 to 2021. Dtsch Ärztebl Int (2022) 119:451–7. 10.3238/arztebl.m2022.0202 35583101 PMC9639227

[4] An Der Heiden M Winklmayr C Buchien S Schranz M Klimawandel RKI-GF Gesundheit DM Wochenbericht Zur Hitzebedingten Mortalität KW (2023). Available online at: https://edoc.rki.de/handle/176904/11310 (Accessed July 2, 2024).

[5] Liss A Naumova EN. Heatwaves and Hospitalizations due to Hyperthermia in Defined Climate Regions in the Conterminous USA. Environ Monit Assess (2019) 191(S2):394. 10.1007/s10661-019-7412-5 31254102

[6] Wang Y Bobb JF Papi B Wang Y Kosheleva A Di Q Heat Stroke Admissions During Heat Waves in 1,916 US Counties for the Period from 1999 to 2010 and Their Effect Modifiers. Environ Health (2016) 15(1):83. 10.1186/s12940-016-0167-3 27503399 PMC4977899

[7] Matthies F Bickler G Cardeñosa Marín N Hales S. Heat–Health Action Plans: Guidance. Copenhagen: World Health Organization. Regional Office for Europe (2008). Available online at: https://iris.who.int/handle/10665/107888 (Accessed September 02, 2024).

[8] Wibowo R Do V Quartucci C Koller D Daanen HAM Nowak D Effects of Heat and Personal Protective Equipment on Thermal Strain in Healthcare Workers: Part B—Application of Wearable Sensors to Observe Heat Strain Among Healthcare Workers Under Controlled Conditions. Int Arch Occup Environ Health (2024) 97(1):35–43. 10.1007/s00420-023-02022-2 37947815 PMC10791845

[9] World Health Organization. Strengthening Response to Pandemics and Other Public-Health Emergencies: Report of the Review Committee on the Functioning of the International Health Regulations (2005) and on Pandemic Influenza (H1N1) 2009. Geneva: World Health Organization (2011). Available online at: https://iris.who.int/handle/10665/75235 (Accessed February 8, 2024).

[10] Gauer R Meyers BK. Heat-Related Illnesses. Am Fam Physician (2019) 99(8):482–9. 30990296

[11] Xu Z Watzek JT Phung D Oberai M Rutherford S Bach AJE. Heat, Heatwaves, and Ambulance Service Use: A Systematic Review and Meta-Analysis of Epidemiological Evidence. Int J Biometeorol (2023) 67(10):1523–42. 10.1007/s00484-023-02525-0 37495745 PMC10457246

[12] Arsad FS Hod R Ahmad N Ismail R Mohamed N Baharom M The Impact of Heatwaves on Mortality and Morbidity and the Associated Vulnerability Factors: A Systematic Review. Int J Environ Res Public Health (2022) 19(23):16356. 10.3390/ijerph192316356 36498428 PMC9738283

[13] Bund/Länder Ad-hoc Arbeitsgruppe Gesundheitliche Anpassung an die Folgendes Klimawandels (GAK). Handlungsempfehlungen Für Die Erstellung Von Hitzeaktionsplänen Zum Schutz Der Menschlichen Gesundheit. Bundesgesundheitsblatt - Gesundheitsforschung - Gesundheitsschutz (2017) 60(6):662–72. 10.1007/s00103-017-2554-5 28492969

[14] Winklmayr C an der Heiden H. Hitzebedingte Mortalität in Deutschland 2022. Epid Bull (2022) 42:3–9. 10.25646/10695.3

[15] UK Health Security Agency, Department of Health and Social Care. Heatwave Plan for England (2022). Report No.: GOV-12960.

[16] Nishimura T Rashed EA Kodera S Shirakami H Kawaguchi R Watanabe K Social Implementation and Intervention with Estimated Morbidity of Heat-Related Illnesses from Weather Data: A Case Study from Nagoya City, Japan. Sustain Cities Soc (2021) 74:103203. 10.1016/j.scs.2021.103203

[17] Thiel J Seim A Grummt S Nesterow I Penesch F Sedlmayr M Tools for Optimizing Healthcare Resource Allocation in Response to Climate Impacts and Heat Action Planning. J Public Health (2024). 10.1007/s10389-024-02357-1

[18] Krefting D Bavendiek U Fischer J Marx G Molinnus D Panholzer T Die Digitalen Fortschrittshubs Gesundheit – Gemeinsame Datennutzung Über Die Universitätsmedizin Hinaus. Bundesgesundheitsblatt - Gesundheitsforschung - Gesundheitsschutz (2024) 67(6):701–9. 10.1007/s00103-024-03883-9 38753021 PMC11166775

[19] Günster C Schmuker C. Gesundheit Und Klimawandel – Welche Potenziale Haben Versorgungsnahe Daten? Bundesgesundheitsblatt - Gesundheitsforschung - Gesundheitsschutz (2024) 67(2):155–63. 10.1007/s00103-023-03828-8 38240844 PMC10834614

[20] Moda HM Filho WL Minhas A. Impacts of Climate Change on Outdoor Workers and Their Safety: Some Research Priorities. Int J Environ Res Public Health (2019) 16(18):3458. 10.3390/ijerph16183458 31533360 PMC6765781

[21] Liu J Varghese BM Hansen A Borg MA Zhang Y Driscoll T Hot Weather as a Risk Factor for Kidney Disease Outcomes: A Systematic Review and Meta-Analysis of Epidemiological Evidence. Sci Total Environ (2021) 801:149806. 10.1016/j.scitotenv.2021.149806 34467930

[22] Meadows J Mansour A Gatto MR Li A Howard A Bentley R. Mental Illness and Increased Vulnerability to Negative Health Effects from Extreme Heat Events: A Systematic Review. Psychiatry Res (2024) 332:115678. 10.1016/j.psychres.2023.115678 38150812

[23] Cheng J Xu Z Bambrick H Prescott V Wang N Zhang Y Cardiorespiratory Effects of Heatwaves: A Systematic Review and Meta-Analysis of Global Epidemiological Evidence. Environ Res (2019) 177:108610. 10.1016/j.envres.2019.108610 31376629

[24] Augustin J Hischke S Hoffmann P Castro D Obi N Czerniejewski A Auswirkungen Thermischer Belastungen Auf Die Gesundheit – Eine Bundesweite Analyse Auf Grundlage Von GKV-Routinedaten Zwischen 2012–2021. Bundesgesundheitsblatt - Gesundheitsforschung - Gesundheitsschutz (2024) 68:119–29. 10.1007/s00103-024-03968-5 39446174 PMC11774979

[25] Klauber H Koch N. Individuelle Und Regionale Risikofaktoren Für Hitzebedingte Hospitalisierungen Der Über 65-Jährigen in Deutschland. In: Günster C Klauber J Robra BP Schmuker C Schneider A, editors. Versorgungs-Report: Klima Und Gesundheit. Medizinisch Wissenschaftliche Verlagsgesellschaft (2021). p. 63–78. Available online at: https://www.mwv-open.de/site/chapters/e/10.32745/9783954666270-5/ (Accessed February 12, 2025).

[26] Page MJ McKenzie JE Bossuyt PM Boutron I Hoffmann TC Mulrow CD The PRISMA 2020 Statement: An Updated Guideline for Reporting Systematic Reviews. BMJ (2021) n71:n71. 10.1136/bmj.n71 33782057 PMC8005924

[27] Ouzzani M Hammady H Fedorowicz Z Elmagarmid A. Rayyan—A Web and Mobile App for Systematic Reviews. Syst Rev (2016) 5(1):210. 10.1186/s13643-016-0384-4 27919275 PMC5139140

[28] Barletta JF Palmieri TL Toomey SA Harrod CG Murthy S Bailey H. Management of Heat-Related Illness and Injury in the ICU: A Concise Definitive Review. Crit Care Med (2024) 52(3):362–75. 10.1097/CCM.0000000000006170 38240487

[29] Faurie C Varghese BM Liu J Bi P. Association Between High Temperature and Heatwaves with Heat-Related Illnesses: A Systematic Review and Meta-Analysis. Sci Total Environ (2022) 852:158332. 10.1016/j.scitotenv.2022.158332 36041616

[30] Kunda JJ Gosling SN Foody GM. The Effects of Extreme Heat on Human Health in Tropical Africa. Int J Biometeorol (2024) 68(6):1015–33. 10.1007/s00484-024-02650-4 38526600 PMC11108931

[31] Levi M Kjellstrom T Baldasseroni A. Impact of Climate Change on Occupational Health and Productivity: A Systematic Literature Review Focusing on Workplace Heat. Med Lav (2018) 109(3):163–79. 10.23749/mdl.v109i3.6851 29943748 PMC7689800

[32] Moon J. The Effect of the Heatwave on the Morbidity and Mortality of Diabetes Patients; A Meta-Analysis for the Era of the Climate Crisis. Environ Res (2021) 195:110762. 10.1016/j.envres.2021.110762 33515577

[33] Noakes T. Fluid and Electrolyte Disturbances in Heat Illness. Int J Sports Med (1998) 19(S 2):S146–9. 10.1055/s-2007-971982 9694423

[34] Santelli J Sullivan JM Czarnik A Bedolla J. Heat Illness in the Emergency Department: Keeping Your Cool. Emerg Med Pract (2014) 16(8):1–22. 25422847

[35] Wu WJ Hutton J Zordan R Ranse J Crilly J Tutticci N Review Article: Scoping Review of the Characteristics and Outcomes of Adults Presenting to the Emergency Department During Heatwaves. Emerg Med Australas (2023) 35(6):903–20. 10.1111/1742-6723.14317 37788821

[36] Haddaway NR Page MJ Pritchard CC McGuinness LA. *PRISMA2020*: An R Package and Shiny App for Producing PRISMA 2020‐Compliant Flow Diagrams, with Interactivity for Optimised Digital Transparency and Open Synthesis. Campbell Syst Rev (2022) 18(2):e1230. 10.1002/cl2.1230 36911350 PMC8958186

[37] Sohn KH Song WJ Park JS Park HW Kim TB Park CS Risk Factors for Acute Exacerbations in Elderly Asthma: What Makes Asthma in Older Adults Distinctive? Allergy Asthma Immunol Res (2020) 12(3):443–53. 10.4168/aair.2020.12.3.443 32141258 PMC7061162

[38] Wang X Chien WT Chong YY. Effectiveness of Psychosocial Interventions for Improving Asthma Symptoms and Parental Stress in Families of School-Age Children with Asthma: A Systematic Review and Meta-Analysis. Int J Nurs Stud (2024) 160:104905. 10.1016/j.ijnurstu.2024.104905 39316993

[39] Botero D Wolfsdorf JI. Diabetes Mellitus in Children and Adolescents. Arch Med Res (2005) 36(3):281–90. 10.1016/j.arcmed.2004.12.002 15925018

[40] Jesdale BM Morello-Frosch R Cushing L. The Racial/Ethnic Distribution of Heat Risk–Related Land Cover in Relation to Residential Segregation. Environ Health Perspect (2013) 121(7):811–7. 10.1289/ehp.1205919 23694846 PMC3701995

[41] Yin Y He L Wennberg PO Frankenberg C. Unequal Exposure to Heatwaves in Los Angeles: Impact of Uneven Green Spaces. Sci Adv (2023) 9(17):eade8501. 10.1126/sciadv.ade8501 37115921 PMC10146884

[42] Watso JC Farquhar WB. Hydration Status and Cardiovascular Function. Nutrients (2019) 11(8):1866. 10.3390/nu11081866 31405195 PMC6723555

[43] Boeckmann M Rohn I. Is Planned Adaptation to Heat Reducing Heat-Related Mortality and Illness? A Systematic Review. BMC Public Health (2014) 14(1):1112. 10.1186/1471-2458-14-1112 25349109 PMC4219109

[44] Stone B Vargo J Liu P Habeeb D DeLucia A Trail M Avoided Heat-Related Mortality Through Climate Adaptation Strategies in Three US Cities. PLoS ONE 9, 6 (2014). 10.1371/journal.pone.0100852 24964213 PMC4071007

[45] Graf S Kranz J Schmidt S Bellut L Uhlig A. Formen Der Evidenzsynthese. Urol (2021) 60(4):434–43. 10.1007/s00120-021-01476-x 33656592 PMC7927776

