# Supplementary material for: The Spectrum of Heat-Related Diseases - A Meta-Review
Source: Int J Public Health. 2025 Sep 16;70:1608592. doi: 10.3389/ijph.2025.1608592 (PMC12479383; doi:10.3389/ijph.2025.1608592)
Supplement: Supplementary file 1 [file Supplementaryfile1.docx]

**Appendix 1 – Search string**

| **Search-Tool** | | **Search string** |
| --- | --- | --- |
| PubMed | ("high temperature stress" OR "heat damage" OR "heat health" OR "heat disease" OR "heat-related disease" OR "heat illness" OR "heat-related illness" OR "heatstroke" OR "heat death")  Filters: German, English, Review, 30 Years | |
| Ovid | ((heat OR high temperature OR heatwave OR heat wave OR heat stroke OR sunstroke OR heat exhaustion OR heat cramps OR heat rash OR heat edema OR heat syncope OR heat illness OR heat injury OR heat related OR heat induced) AND (disease OR illness OR condition OR disorder OR morbidity OR mortality))  Filter:  Embase <1974 to 2024 May 13> Ovid MEDLINE(R) ALL <1946 to May 10, 2024>, 1994-2024, DE EN, human, review | |

**Appendix 2 - Detailed results**

| **Title** | **Year** | **Author** | **Illness** | **Population** | **Climatic influencing factors** | **Other influencing factors** |
| --- | --- | --- | --- | --- | --- | --- |
| Association between high temperature and heatwaves with heat-related illnesses: A systematic review and meta-analysis | 2022 | Faurie C , Varghese B M, Liu J and Bi P | ICD 9:   - 276 - 584 - 992 - 992.3 - 992.5 - 265.5 - 276.5   ICD 10:   - E85 - E86 - E87 - E900 - R50.9 - T67 - X30 - X32 - X54 - T76 | non specific | Temperature, air pressure | Elderly (age > 65 years) |
| Cardiorespiratory effects of heatwaves: A systematic review and meta-analysis of global epidemiological evidence | 2019 | Cheng J, Xu Z, Bambrick H, Prescott V, Wang N, Zhang Y, Su H, Tong S and Hu W | - Ischemic heart stroke - Heart failure - Asthma - Chronic obstructive pulmonary disease (COPD) | non specific | Temperature | Male gender, age (younger and older people) |
| Fluid and electrolyte disturbances in heat illness. | 1998 | Noakes TD | - Heat cramps - Heat exhaustion (heat syncope) - Heat stroke | athletes | Temperature, humidity, wind movement, | Sweating rate, exercise intensity, preexisting dehydration, individual susceptibility |
| Heat illness in the emergency department: keeping your cool. | 2014 | Santelli J, Sullivan J M, Czarnik A and Bedolla J | - Heat stroke - Heat exhaustion - Heat cramps - Heat syncope (fainting) - Heat rash (miliaria) - Hyperthermia - Heat edem - heat tetany | non specific | Temperature, humidity | Drug and alcohol use, absence of adequate breaks, absence of shelter or shade, lack of access to water, age (age < 15 years or age > 65 years), deconditioning or sedentary, excessive clothing, inadequate sleep, lack of acclimatization, large muscle mass, male sex, obesity, preexisting dehydration, cardiac disease, congenital disorder, diabetes mellitus, previous heat injury, recent or acute illness, sickle cell trait, skin abnormalities |
| Heat-Related Illnesses. | 2019 | Gauer R and Meyers BK | - Heat edema - Exercise-associated muscle cramps (heat cramps) - Heat rash (miliaria rubra) - Exercise-associated collapse (heat syncope) - Heat exhaustion - Heat stroke | high school athletes, Armed Forces | Temperature,  (relative) humidity, direct radiation sunlight | Clothing, inadequate sleep, lack of acclimatization, large muscle mass, male sex, obesity, preexisting dehydration  Medical conditions: cardiac disease, congenital disorder (e.g., ectodermal dysplasia, idiopathic  anhidrosis), diabetes mellitus, previous heat injury, recent or acute illness, sickle cell trait, skin abnormalities (e.g., burns, psoriasis, eczema), radiation, homeless people |
| Heat, heatwaves, and ambulance service use: a systematic review and meta-analysis of epidemiological evidence. | 2023 | Xu Z, Watzek JT, Phung D, Oberai M, Rutherford S and Bach AJE | - Heat stroke - Dehydration - Heat exhaustion - Myocardial infarction/ stroke - Acute kidney injury - Out-of-hospital cardiac arrest (OHCA) - Acute coronary syndrome - Respiratory distress | non specific | Temperature, (relative) humidity, | Age (children or age > 65 years), female gender, low socioeconomic status |
| Hot weather as a risk factor for kidney disease outcomes: A systematic review and meta-analysis of epidemiological evidence | 2021 | Liu J,Varghese BM, Hansen A, Borg MA, Zhang Y, Driscoll T, Morgan G, Dear K, Gourley M, Capon A and Bi P. | ICD10:   - Kidney disease (N00-N39) - Kidney failure (N17-N19) - Urolithiasis (N20−N23) - Urinary tract infections (N10-N12, N30, N39) | non specific |  | Male gender, age (age ≤64 years), people living in temperate climate zones, chronic dehydration |
| Impact of climate change on occupational health and productivity: a systematic literature review focusing on workplace heat. | 2018 | Levi M and Kjellstrom T and Baldasseroni A | ICD-9:   - 992   ICD-10:   - T67 - V93.2 - X30 - X32 | non specific | Temperature | Dehydration, preexisting conditions (like chronic kidney disease or mental health issues) |
| Impacts of Climate Change on Outdoor Workers and their Safety: Some Research Priorities. | 2019 | Moda HM, Filho WL and Minhas A | - Heat stress/   stroke   - Fatigue - Dehydration - Kidney disease - Respiratory distress - Vector based diseases | Outdoor workers | Temperature, humidity, air quality, air pollution, air movement | Work environment,  socioeconomic status,  safety practices, cultural factors |
| Management of Heat-Related Illness and Injury in the ICU: A Concise Definitive Review. | 2024 | Barletta JF, Palmieri TL, Toomey SA, Harrod CG, Murthy S and Bailey H | - Heat stroke - Heat exhaustion - Heat syncope - Heat cramps - Heat edema - Heat intolerance - Heat rash | Patient at intensive care unit | Temperature, humidity | Excessive exercise, prolonged exposure to heat,  pre-existing conditions (like cardiovascular disease, diabetes), certain medications,  substance abuse |
| Mental illness and increased vulnerability to negative health effects from extreme heat events: a systematic review | 2024 | Meadows J, Mansour A, Gatto MR, Li A, Howard A and Bentley R | ICD-9:   - 290-299: Psychoses (e.g., schizophrenia, affective psychoses, delusional disorders) - 300.4: Dysthymic disorder - 301.1: Affective personality disorder - 309.0: Adjustment disorder with depressed mood - 309.1: Prolonged depressive reaction - 311: Depressive disorder, not elsewhere classified   ICD-10:   - X30: Exposure to excessive natural heat (hyperthermia) - T67: Heat-related disorders (heatstroke, heat exhaustion, heat cramps) - E86: Volume depletion (dehydration) - F20-F22: Schizophrenia and other primary psychotic disorders - F31: Bipolar disorder - F34: Persistent mood [affective] disorders (e.g., dysthymia, cyclothymia) - F43: Reaction to severe stress, and adjustment disorders | Patient with mental illness | Temperature | Low socioeconomic status, use of psychotropic drugs |
| Review article: Scoping review of the characteristics and outcomes of adults presenting to the emergency department during heatwaves. | 2023 | Wu WJ, Hutton J, Zordan R, Ranse J, Crilly J, Tutticci N, English T and Currie J | - Heat exhaustion - Heat stroke - Heat cramps - Fluid and electrolyte imbalance - Acute kidney injury | non specific | Temperature, humidity | Elderly (age > 65 years), male gender, pre-existing conditions (cardiovascular disease, diabetes, respiratory disorders), low socioeconomic status, certain medications, use of psychotropic drugs, occupational activity without protection from the weather, white skin/population |
| The effect of the heatwave on the morbidity and mortality of diabetes patients; a meta-analysis for the era of the climate crisis | 2021 | Moon J, | - Diabetic ketoacidosis - Hyperosmolar hyperglycemic state - Hypertensive diseases - Ischemic heart disease - Cerebrovascular diseases - Acute kidney injury - Asthma - Exacerbations - Chronic bronchitis - Mood disorders | Diabetes patients | Temperature, humidity, air pollution | Age (age > 50 years), male gender, comorbidities (cardiovascular or respiratory diseases), low socioeconomic status, certain medications, particularly those affecting fluid balance or thermoregulation, physical activity during hot weather, health literacy related to diabetes |
| The effects of extreme heat on human health in tropical Africa. | 2024 | Kunda JJ, Gosling SN and Foody GM | - Diarrhea - Respiratory infections - Malaria - Heat stroke - Heat exhaustion - Heat cramps - Dehydration - Kidney failures | non specific | Temperature, humidity | Low socioeconomic status, age (elderly and children), low urbanization, occupational exposure (particularly outdoor workers) |
| The Impact of Heatwaves on Mortality and Morbidity and the Associated Vulnerability Factors: A Systematic Review | 2022 | Arsad FS, Hod R, Ahmad N, Ismail R, Mohamed N, Baharom M, Osman Y, Radi MFM and Tangang F | - Heat stroke - Asthma - Chronic obstructive pulmonary disease (COPD) - Pneumonia - Renal disease | non specific | Temperature | Age (age > 65, age < 5), people with pre-existing conditions (like mental, cardiovascular disease), low socioeconomic status, low education |
|  |  |  |  |  |  |  |
